# Functional Bidirectionality of ERV-Derived Long Non-Coding RNAs in Humans

**DOI:** 10.3390/ijms251910481

**Published:** 2024-09-29

**Authors:** Yanmei Song, Hongling Wen, Xiuli Zhai, Lei Jia, Lin Li

**Affiliations:** 1 Department of Microbiological Laboratory Technology, School of Public Health, Cheeloo College of Medicine, Shandong University, Key Laboratory for the Prevention and Control of Emerging Infectious Diseases and Biosafety, Jinan 250012, China; songyanmei0507@163.com (Y.S.); wenhongling@sdu.edu.cn (H.W.); 2 State Key Laboratory of Pathogen and Biosecurity, Academy of Military Medical Sciences, Beijing 100850, China; zhaixiuli2021@126.com; 3 Department of Microbiology, School of Basic Medicine, Anhui Medical University, Hefei 230000, China

**Keywords:** endogenous retroviruses, long non-coding RNAs, physiological and pathological regulation, mechanism

## Abstract

Human endogenous retroviruses (HERVs) are widely recognized as the result of exogenous retroviruses infecting the ancestral germline, stabilizing integration and vertical transmission during human genetic evolution. To date, endogenous retroviruses (ERVs) appear to have been selected for human physiological functions with the loss of retrotransposable capabilities. ERV elements were previously regarded as junk DNA for a long time. Since then, the aberrant activation and expression of ERVs have been observed in the development of many kinds of human diseases, and their role has been explored in a variety of human disorders such as cancer. The results show that specific ERV elements play respective crucial roles. Among them, long non-coding RNAs (lncRNAs) transcribed from specific long-terminal repeat regions of ERVs are often key factors. lncRNAs are over 200 nucleotides in size and typically bind to DNA, RNA, and proteins to perform biological functions. Dysregulated lncRNAs have been implicated in a variety of diseases. In particular, studies have shown that the aberrant expression of some ERV-derived lncRNAs has a tumor-suppressive or oncogenic effect, displaying significant functional bidirectionality. Therefore, theses lncRNAs have a promising future as novel biomarkers and therapeutic targets to explore the concise relationship between ERVs and cancers. In this review, we first summarize the role of ERV-derived lncRNAs in physiological regulation, mainly including immunomodulation, the maintenance of pluripotency, and erythropoiesis. In addition, pathological regulation examples of their aberrant activation and expression leading to carcinogenesis are highlighted, and specific mechanisms of occurrence are discussed.

## 1. Introduction to ERVs

### 1.1. Origins and Evolution of Early ERVs

At the beginning of the 21st century, the first large-scale sequencing and analysis of the human genome revealed that 45% of the human genome is composed of transposable elements (TEs) [1]. Endogenous retroviruses (ERVs) are retrotransposons, a type of TE, and symbolize the remnants of ancient germline cell infections by exogenous retroviruses [2]. Most ERVs entered the germline genome of ancient ancestors via retroviral infection over 30 million years ago and accumulated mutations at a natural rate [3]. Subsequently, they spread horizontally throughout the genome via a copy-and-paste mechanism on one hand [4,5], and are vertically transmitted to the descendants according to Mendelian law on the other hand [2,5]. Taking the human genome as an example, the reference genome assembly contains approximately 450,000 ERV-derived elements that can be stratified into nearly 100 families, accounting for around 8% of the whole DNA [1]. All ERV families discovered in humans were subsequently identified in other primates, except for some very young ERV loci that are not conserved in other species [5,6,7]. Up until now, ERVs have continued to evolve [8]. In addition, ERV-derived elements and proteins play important roles in resisting exogenous viral infections, regulating embryonic trophoblast development and carcinogenesis [9,10,11].

### 1.2. Classification and Structure of ERVs

Approximately 8% of the whole human genome consists of ERVs [12]. The classification of human endogenous retroviruses (HERVs) is complex, with different classification systems coexisting. An early system was based on the tRNA molecule used by retroviruses as a primer in replication. The primer binding site (PBS) regions of Class II are complementary to lysine (K) tRNA molecules, designated HERV-K [13]. Among the variety of human endogenous retroviral families, HERV-K was the latest acquired by the human species [14]. HERV-K is the most complete and biologically active family in the human genome and is closely related to many cancers and neurological diseases [15]. HERV-K is divided into subfamilies, from HML-1 through HML-10. These proviruses appeared about 30~35 million years ago [16,17].

In addition to the classification system above, HERVs have been divided into three classes based on phylogenetic analysis, of which Class I consists of Gamma retrovirus-like elements, Class II of Beta retrovirus-like elements, and Class III of vaguely Spuma retrovirus-like elements [18]. 

ERVs are the inheritance of exogenous retroviral infections from ancestral germ cell lineages, and they have the same internal structure as exogenous retroviruses such as HIV [12]. The complete ERV genomic structure consists of flanking long terminal repeats (LTRs) and internal regions of *gag*, *pro*, *pol*, and *env* genes encoding the structural and functional proteins of the virus that are essential for viral replication [18]. The *gag* gene encodes capsid (CA), nucleocapsid (NC), and matrix (MA) proteins; the *pro* gene encodes proteases; the *pol* gene encodes reverse transcriptases (RTs) and integrases (INs); and the *env* gene encodes surface and transmembrane proteins [19,20,21]. The 5′ and 3′ LTRs are identical and located at both ends of an ERV provirus. The 5′ LTR contains transcriptional regulatory sequences with numerous promoter and enhancer binding sites [19,22]. During human genetic evolution, almost all ERV elements have lost their retrotransposable potential with the accumulation of genetic variation, with only proviral fossils left [17,23,24,25]. In addition, ERVs undergo frequent genetic recombination and deletion, and the genetic recombination between flanking 5′ and 3′ LTRs causes the abundant formation of solo LTR elements [26]. Human ERVs (HERVs) have been present in the human genome for tens of millions of years, and approximately 90% of these HERVs are solo LTR elements [27].

### 1.3. Activation of ERVs in Health and Disease

ERVs have established long-term interrelationships with their hosts over millions of years of evolution, and some ERVs have been selected by their hosts during the evolutionary process to play normal regulatory roles in genes and gene networks. For example, increasing evidence indicates that human ERVs can deeply affect human health and disease [5,28,29,30,31].

ERV elements have been discovered to play critical roles in embryonic development, neural development, and antiviral infections. ERVs mainly function in two ways. One way is based on provirus-encoded protein that acts in reverse on the host, while the other is based on LTR elements that can exert transcriptional regulatory potential to interact with the expression of upstream and downstream genes. A typical physiological function of HERVs is their involvement in the formation of the placenta [32]. A study conducted by Lu et al. revealed that HERVH can maintain the identity of human embryonic stem cells (hESCs) [33]. High levels of HERV-K (HML-2) transcripts and proteins have been identified in undifferentiated embryonic stem cells (ESCs) and induced pluripotent stem cells (iPSCs). The induction of differentiation silences their expression very rapidly [34]. In addition, Syncytin-1 and 2 are the Env proteins of HERV-W and HERV-FRD, respectively, which maintain the fusion trophoblast cell layer and its connection with the cytotrophoblast layer [35]. Subsequently, Frank et al. characterized an envelope-derived protein, Suppressyn, among a large pool of envelope-derived sequences within the human genome to test the potential to restrict retroviral infection. The results found that Suppressyn is expressed in human preimplantation embryos and the developing placenta using its ancestral retroviral promoter. In vitro cell culture assays revealed that Suppressyn and its hominoid orthologs can restrict infection by extant mammalian type D retroviruses [9]. In addition, as has been observed in the pluripotent cells of early embryos, placental tissues, the immune system, and other biological contexts, ERVs contribute significantly to gene regulatory innovation [5]. 

Conversely, the aberrant activation of ERVs also affects the development of diseases, such as neurodegenerative disorders, immune disorders, and cancer [36]. A study reported the increased transcription of HERV-W elements, as well as the presence of antigens of HERV-W envelope and capsid proteins, in blood samples from schizophrenia patients compared to healthy controls [37]. In addition, significantly higher HERV-K10 activity was detected in the brains of schizophrenic patients compared to healthy controls [38]. Furthermore, ERVs are considered pathogens of autoimmune diseases because of their similarity to the structure and sequence of exogenous retroviruses associated with immune dysregulation. Indeed, retrovirus-like particles and immune responses to ERV proteins, distinct from known exogenous retroviruses, have been observed in autoimmune diseases, and ERVs are a factor in the pathogenesis of systemic lupus erythematosus [39]. ERVs may lead to immune dysregulation due to the insertion of mutagens or cis-regulatory elements in cellular genes involved in immune function. ERVs can also encode elements capable of activating cellular genes in trans, such as tax in human T-lymphotropic virus type I (HTLV-I) or tat in human immunodeficiency virus I (HIV-I) [40]. In particular, ERVs play a potential role in cancer by leading to the aberrant activation and expression of oncogenes. There is considerable evidence in ERV-induced cancer that Rec and Np9 are HERV-K-encoded helper proteins that induce intercellular fusion and promote tumorigenesis, and are abundant in tumorized germ cells and virtually absent in healthy germ cells [41,42]. In addition, the LTR of ERV contains regulatory sequences for many host genes and is a rich natural promoter library in the human genome [43], so it is not surprising that aberrant transcriptional activation of the LTR leads to cancer development. Li et al. found that LTR-transcribed lncRNAs influence tumorigenesis, and HERV-K11-derived ncRNA binds directly to the repressor of the proto-oncogene transcription of PSF proteins to drive human tumorigenesis [44].

## 2. Introduction to LncRNAs

### 2.1. Definition and Classification of LncRNAs

**Figure 1:**
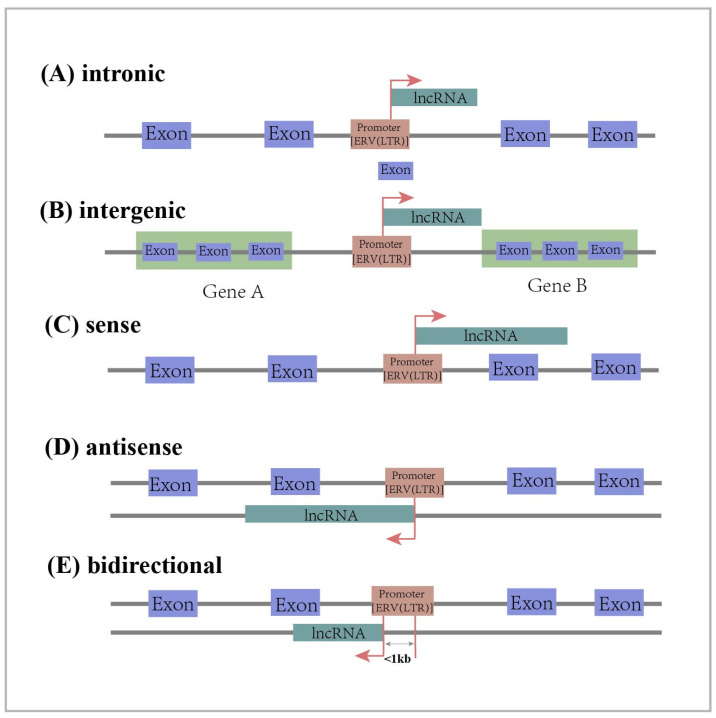
Classification of lncRNAs according to their position in relation to the genome: intronic lncRNA, long intergenic lncRNA, sense lncRNA, antisense lncRNA, bidirectional lncRNA. The red arrow represents the transcription direction of lncRNA.

Long non-coding RNAs (lncRNAs) are RNAs longer than 200 nt that do not encode proteins [45,46]. They belong to a large family of RNAs that are generally classified into five categories based on their location on chromosomes relative to the transcription sites of protein-coding genes. Intronic lncRNA is transcribed from the intronic regions of protein-coding genes. Long intergenic ncRNA (lincRNA) is transcribed from the intermediate region of two protein coding genes. Sense lncRNA is transcribed from the sense strand of a protein-coding gene and overlaps one or more exons of the protein-coding gene. Antisense lncRNA is transcribed from the antisense strand of a protein-coding gene and overlaps one or more exons of the protein-coding gene. Bidirectional lncRNA shares the same promoter as the protein-coding gene, but is transcribed in the opposite direction from within 1 kb of the promoter region of the protein-coding gene (Figure 1) [45,46,47]. As regulatory factors, lncRNAs play an important role in cellular processes such as regulating cell growth and differentiation [48,49,50]. In addition, they also have an impact on the development of diseases. Some studies have confirmed that lncRNAs act as oncogenes or tumor suppressors in the development of cancer by regulating the proliferation, migration, and invasion of cancer cells [51,52].

### 2.2. Sources of LncRNAs

Progress has been made in understanding the origin and evolution of lncRNAs, and transposable element (TE) insertion has been found to be an important mode of lncRNA evolution [53]. In vertebrates, TEs occupy a large portion of the genome, most of which are transposed with the help of intermediate RNAs, mainly including endogenous retroviruses (ERVs), long interspersed nuclear elements (LINEs), short interspersed nuclear elements (SINEs), and DNA transposons [54,55,56]. In addition, TEs carry cis-regulatory elements such as promoters [57,58], enhancers [59], and transcription factor binding sites [60,61,62,63]. Further assessment of the impact of TEs on the cis-regulation of lncRNA genes revealed that TEs affect lncRNA transcription [64]. However, is the contribution of all TEs to lncRNAs the same? Kapusta and colleagues found TE enrichment in the first exon and upstream regions of human lncRNAs, and statistical analysis revealed that LTR/ERV contributed the most compared to other types of TEs [65]. The systematic assessment of the study suggests that ERV/LTRs are important players in the origin, diversification, and regulation of lncRNAs. 

Surprisingly, there were studies that analyzed the contribution of TEs to lincRNAs in detail. Cohen et al. characterized the distribution of TEs in the human genome, indicating that lincRNAs contain more TE sequences relative to protein-coding genes, and there is considerable ERV enrichment at TE positions in lincRNAs. In contrast, the other two major families, LINE and SINE, are barely enriched in lincRNAs [66]. Interestingly, TEs, and especially ERV elements, shape pluripotency networks mainly through lncRNAs’ regulatory systems [67]. For example, Loewer and colleagues isolated induced pluripotent stem cells (iPSCs) using cellular reprogramming and identified a set of pluripotency-associated lincRNAs. Ten lincRNAs were found to be highly expressed in iPSCs compared to embryonic stem cells (ESCs), and seven of these lincRNA genes had HERVH elements on them, suggesting that HERVH-derived lincRNAs play a critical role in iPSCs [68]. However, how does the HERV regulate lincRNA transcription? Cohen et al. further explored the position and orientation of TEs at the lincRNA gene locus. The results suggest that the ERV LTR peaked in coverage at the transcription start site (TSS) of lincRNAs and was more oriented in the lincRNA-positive direction, whereas the LINE L1 and SINE Alu families did not have a peak in coverage at the TSS of lincRNAs [66]. Based on the correlation between ERV LTR and lincRNA TSS, it can be inferred that there are important regulators in the ERV LTR region that regulate lincRNA transcription [69,70]. Overall, the above two studies complement each other by studying different lncRNA sets to reach a common conclusion that TEs, and especially ERVs, are involved in lncRNA derivation, influencing genome regulation and shaping new ways to evolve.

### 2.3. Functional Mechanisms of lncRNAs

Currently, there is growing evidence that lncRNAs are able to interact with nucleic acids (DNA, RNA) and proteins and participate in a wide range of biological functions through different molecular mechanisms, including gene expression regulation [71], cellular differentiation, and disease genesis [72]. Therefore, understanding the biological roles of lncRNAs will contribute to our understanding of this frontier of molecular biology. lncRNAs act at multiple levels from epigenetic and transcriptional regulation to post-transcriptional regulation [73].

Firstly, lncRNAs are involved in epigenetic regulation, which is generally considered a genetic mechanism in which the sequence of DNA is not altered while the expression of genes is altered by regulation at the chromatin level [74]. It has been reported that lncRNA silences genes by mediating chromatin-remodeling complexes, especially the regulation of some histone methyltransferases associated with histone modifications. For example, Polycomb Repressive Complex 2 (PRC2), a common methyltransferase of H3K27, can be regulated by lncRNA HOTAIR, causing the methylation of histone H3K27. This methylation allows chromatin to form a heterochromatin state, with changes in chromatin openness causing the silencing of gene expression [75]. In addition, in terms of transcriptional regulation, lncRNAs can modulate the regulatory activity of transcription factors to affect gene expression. For example, RNA polymerase II is involved in the transcription of genes, and although lncRNA is also transcribed by RNA polymerase II, post-transcriptional lncRNA may bind to RNA polymerase II to affect the transcriptional activity of RNA polymerase II [76]. lncRNAs can also bind to RNA-binding proteins (RBPs), thereby affecting gene expression. It has been reported that lncRNA HBBP1 is essential for erythropoiesis by upregulating TAL1, a key regulator of erythropoiesis, through the binding protein HNRNPA1 [77]. In addition to interacting with RBP, lncRNAs can also interact directly with DNA to produce hybrid structures that affect chromatin accessibility. Two forms of interaction have been proposed, the RNA–DNA–DNA triplex and the R-loop, in which lncRNA binds to DNA to regulate the transcription of target motifs via cis or trans [78]. 

Splicing regulation and translational control are two common post-transcriptional regulatory mechanisms for lncRNAs. Most protein-coding genes are composed of exons and introns, which are transcribed to generate pre-mRNA. Variable splicing generates multiple mRNA splice variants from a single pre-mRNA, which increases the diversity of the transcriptome and the proteome and is an important step in the regulation of gene expression in mammalian cells. Splicing takes place mainly in the spliceosome of the nucleus. The spliceosome consists mainly of five small nuclear ribonucleoprotein particles (snRNPs), namely U1, U2, U4, U5, and U6, and non-snRNP splicing factors [79,80]. Another U3 snRNA is associated with the maturation of 28S rRNA in the nucleolus and is involved in ribosomal RNA processing [81]. There are various mechanisms of lncRNAs that affect gene splicing. The first is that lncRNA binds to splicing factors to block the formation of spliceosome complexes. For example, the myocardial-infarction-associated transcript, lncRNA *MIAT*, contains tandem UACUAAC repeats, binds to SF1 splicing factors with high affinity and is able to inhibit splicing and spliceosome complex formation [82]. Secondly, it has also been reported that lncRNAs regulate the modification of splicing factors (e.g., phosphorylation) and that the metastasis-associated lung adenocarcinoma transcript lncRNA *MALAT1* affects the alternative splicing of pre-mRNAs by modulating SR splicing factor phosphorylation [83]. In addition, the lncRNA binds to intronic regions to inhibit splicing factor binding [47]. Regarding translational regulation, lncRNAs act by binding to translation factors or ribosomes. Parrott et al. reported that a cytoplasmic lncRNA *snaR* associated with nuclear factor 90 (NF90/ILF3) proteins binds to ribosomes and affects the translation of mRNAs [84]. In addition, for post-transcriptional regulation, lncRNAs act as microRNA (miRNA) sponges or decoys to attract miRNAs and competitively isolate them from target mRNAs. A central premise of this regulatory mechanism is that lncRNAs and a particular mRNA share a common binding miRNA, resulting in the inability of the miRNA to bind to the mRNA, thereby affecting the translation and expression of the mRNA [85]. In summary, lncRNAs have multiple functions in the cell. They play key roles in epigenetic, transcriptional, and post-transcriptional regulation; however, research on the mechanism of lncRNA function is a rapidly developing field, and much is not yet fully understood.

ERV-derived lncRNAs play important roles in gene regulation and various biological processes, resisting exogenous viral infections, maintaining the normal physiological functions of the immune system, regulating hESCs and early embryonic networks, maintaining pluripotency, and regulating erythropoiesis [86,87,88]. When ERVs are inappropriately activated, LTR-derived lncRNAs also contribute to the development of a variety of diseases. This article focuses specifically on the involvement of lncRNA dysregulation in the development of a variety of cancers, such as hepatocellular carcinoma, breast cancer, and melanoma. By describing the interactions between ERV-derived lncRNAs and the host during the evolutionary process, we are able to gain a deeper understanding of the relationship between lncRNAs in the normal physiological regulation of an organism, as well as in the development of diseases, especially cancers, which can make an important contribution to the prevention and treatment of diseases [5,89].

## 3. ERV-Derived lncRNAs Involved in Physiological Regulation

### 3.1. Immune Regulation

There is increasing evidence that ERVs contribute to the maintenance of the normal physiological function of the host immune system against exogenous viruses [90]. In the host antiviral immune response, pathogens are recognized by host pattern recognition receptors (PRRs), which mainly include Toll-like receptors (TLRs), nucleotide oligomerization domain (NOD)-like receptors (NLRs), retinoic-acid-inducible gene-I (RIG-I)-like receptors (RLRs), C-type lectin receptors (CLRs), and absent in melanoma-2 (AIM2)-like receptors (ALRs) [91]. Then, downstream transcription factors are activated, such as interferon regulatory factors and NF-κB transcription factors, which can upregulate the expression of genes involved in the inflammatory response, induce the production of type I interferons (IFNs), and promote the transcription of interferon-stimulated genes (ISGs) as cellular antiviral defenders [92].

**Figure 2:**
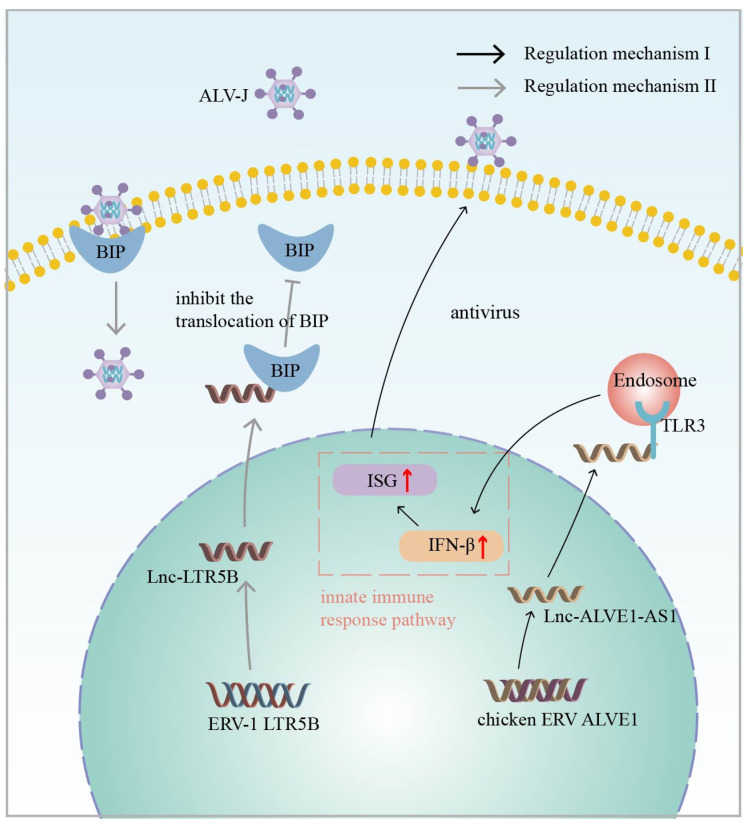
Regulatory mechanism I (Black Arrow): Chicken ERV-derived lnc-*ALVE1-AS1* positively regulates IFN-β, a key gene in the type I interferon (IFN) pathway, and interferon-stimulated genes (ISGs) through signaling of the cytoplasmic sensor Toll-like receptor 3 (TLR3) to induce antiviral innate immunity. Regulatory mechanism II (Grey Arrow): Chicken ERV-derived lnc-*LTR5B* interacts with binding immunoglobulin protein (BIP) and inhibits BIP ectopic to the cell surface, which has an inhibitory effect on avian leukosis virus subgroup J (ALV-J) replication. The red arrow represents upregulation of expression.

Thus, ERV-derived lncRNAs play a critical role during the immune process against exogenous viral infections. Previously, the endogenous avian leukosis virus in chromosome 1 (ALVE1) was found to transcribe a lncRNA, lnc-*ALVE1-AS1*, that can inhibit exogenous virus replication in chicken embryonic fibroblasts (CEFs), protecting them from exogenous viruses. Mechanistically, the ERV-derived lnc-*ALVE1-AS1* triggers PRR and upregulates the expression of the cytoplasmic sensor gene TLR3. This, in turn, positively regulates IFN-β, a key gene in the type I interferon pathway, and ISGs protects CEFs from exogenous viral infections (Figure 2: Regulatory mechanism I) [93,94]. Interestingly, it was also found that lnc-*ALVE1-AS1* not only induces an antiviral immune response in non-immune cells, but also has a similar function in immune cells [95]. In addition to this, Chen et al. identified a lncRNA derived from ERV-LTR in chickens, named lnc-*LTR5B*. During avian leukosis virus subgroup J (ALV-J) infection, lnc-*LTR5B* expression is reduced and induces the translocation of binding immunoglobulin protein (BiP) to the cell surface. Conversely, the overexpression of lnc-*LTR5B* promotes the binding of lnc-*LTR5B* to BiP and exerts an inhibitory effect on ALV-J replication (Figure 2: Regulatory mechanism II) [94]. The above studies have demonstrated that endogenous retrovirus-derived lncRNAs play an important role in resistance to exogenous viral infections and provide new strategies for vaccine development [96].

**Figure 3:**
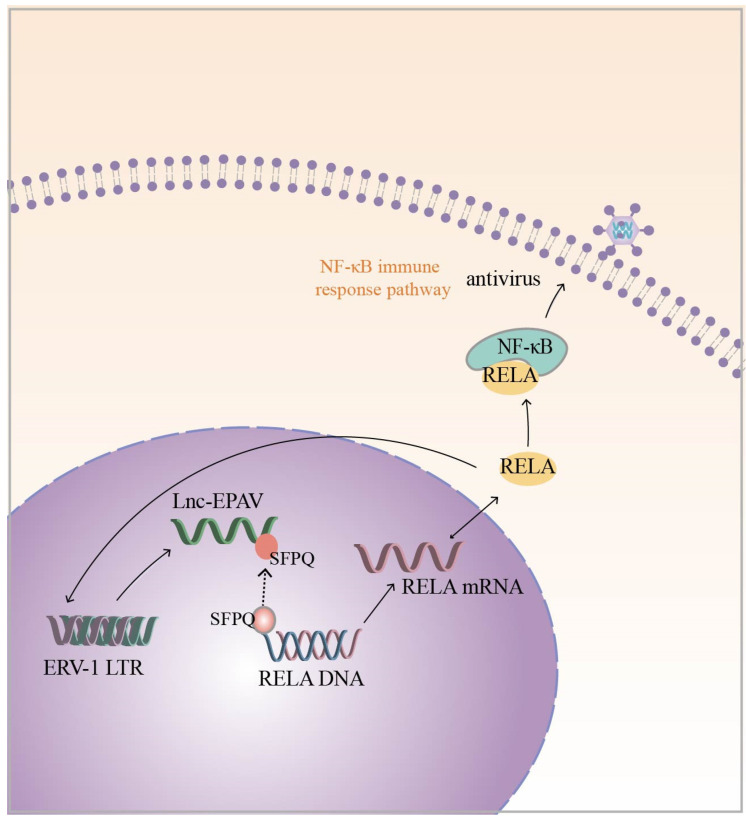
Infection of a host with pathogenic mimics leads to upregulation of ERV-1 LTR-derived lnc-*EPAV* expression. lnc-*EPAV* competitively binds to splicing factor proline- and glutamine-rich (SFPQ) (The black dashed arrow represents the dynamic), a transcriptional repressor of immunogen *RELA*, which dissociates SFPQ from the *RELA* promoter and promotes *RELA* expression. This, in turn, positively regulates NF-κB signaling and promotes the innate immune response. *RELA*, in turn, influences the transcriptional activation of lnc-EPAV (*RELA* is a subunit of NF-κB). The black solid arrow represents the regulatory mechanism.

In addition to the above chicken endogenous retroviruses, Zhou et al. also identified a novel lncRNA derived from the ERV1 LTR, namely lnc-*EPAV*, in mouse macrophages with a potential role in the antiviral innate immune response. The silencing of lnc-*EPAV* with shRNA in mouse macrophages under viral infection conditions resulted in an increased ability of the virus to replicate in the cells. In in vivo experiments, lnc-*EPAV*-deficient mice with reduced serum levels of IFN expression showed increased susceptibility to viral infection [86]. These results indicate that lnc-*EPAV* may act as a modulator of the antiviral immune response. Regarding the specific mechanism, the infection of the host by pathogenic mimics leads to the upregulation of lnc-*EPAV* expression, which, in turn, promotes *RELA* expression. NF-κB plays a critical role in the antiviral immune response, and *RELA* is a subunit of NF-κB [97,98]. The effect of lnc-*EPAV* on *RELA* transcriptional expression is mainly dependent on SFPQ, which is a transcriptional repressor of the immunogen *RELA*. Under viral infection conditions, lnc-*EPAV* competitively binds SFPQ, which dissociates SFPQ from the *RELA* promoter and promotes *RELA* expression. Interestingly, lnc-*EPAV* positively regulates *RELA* expression, and *RELA*, in turn, affects the transcriptional activation of lnc-*EPAV*, forming a positive feedback loop (Figure 3). Overall, lnc-*EPAV* positively regulates NF-κB signaling and promotes innate immune responses by binding to repressors of immune genes [86]. In addition to the ERV1-derived lncRNAs mentioned above, several other representative HERV-derived lncRNAs, such as *MER9a2*, *MLT2A1*, and *LTR5A*, were found to cooperate with SFPQ to promote host antiviral immunomodulation [86]. Although lncRNAs are not conserved during evolution, it is currently hypothesized that ERV-derived lncRNA and SFPQ interactions are universal and play important biological roles in antiviral immune regulation.

### 3.2. Pluripotency Regulation

There is growing evidence that ERV-derived lncRNAs are important regulators of pluripotency development. Durruthy-Durruthy and colleagues identified ERV-derived lncRNAs (*HPAT2*, *HPAT3*, *HPAT5*) as transcripts associated with human pluripotency that are specifically expressed in the inner cell mass (ICM) and maintain the pluripotent state of human embryonic stem cells (hESCs) [99]. In particular, *HPAT5*, a key component of the core regulatory network for pluripotency, consists of SINE and HUERS-P1 LTR elements. *HPAT5* is regulated by NANOG, a key transcription factor associated with pluripotency. Based on transcriptome and protein analyses, *HPAT5* was found to maintain the identity of the cellular pluripotent state by inhibiting the let7 family of microRNAs towards maturation [99].

In addition, Lu et al. targeted HERVH with short hairpin RNA (shRNA) to investigate the role of HERVH in hESCs. Interestingly, a shift in cell morphology towards fibrous was observed, with the downregulation of pluripotency markers (OCT4, SOX2, NANOG) and the upregulation of differentiation markers (GATA6, RUNX1) [33]. Further analysis revealed that HERVH LTR gene regions transcribe lncRNAs, and these regions bind the pluripotency-related transcription factor OCT4, which regulates lncRNA transcription [100]. Overall, lncRNAs from HERVH play the role of maintaining the pluripotent state of hESCs.

The transcriptional and epigenetic regulatory network of hESCs is controlled by multiple regulatory circuits, starting with core transcription factors (TFs) such as OCT4, SOX2, and NANOG, which maintain hESCs’ self-renewal [101]. Then, miRNAs, which have been identified as important post-transcriptional modifiers, are involved in the direct repression of TFs [102]. In addition, there are potential mechanisms for lncRNAs in the hESC regulatory network, especially in the TF and miRNA linkage network. What kind of correlations exist between the numerous regulatory circuits of hESCs? Wang found that lincRNA regulates TF expression in hESCs, and, at the same time, TF expression affects lincRNA transcription, suggesting that TF and lincRNA form a regulatory feedback loop in hESC. lincRNA (linc-*ROR*) is essential for the regulation of hESCs’ self-renewal. linc-*ROR* functions as a competitive endogenous RNA (ceRNA) that shares miRNA elements with TF and acts as a sponge for miRNAs, preventing TF from being inhibited by miRNAs [103]. It has been shown that linc-*ROR* acts as a miRNA sponge, reducing the effective concentration of miR-145 and preventing the removal of OCT4, SOX2, and NANOG-transcribed mRNAs [102]. Thus, lncRNAs and TFs, as well as miRNAs, form a closed regulatory loop that regulates hESC pluripotency and differentiation.

### 3.3. Erythropoiesis Regulation

Erythropoiesis is an essential process in mammalian development. In fetal liver and adult bone marrow, pluripotent stem cells differentiate into hematopoietic cells, and many transcription factors and non-coding RNAs influence erythropoiesis [104]. Several studies have been conducted to characterize the effect of lncRNAs on erythropoiesis [105,106,107], which plays a crucial role in the search for treatments for erythrocytic diseases. There are approximately 4000 ERV-9 LTR copies in the human genome that do not contain the internal ERV genes, the major structures of which are U3, R, and U5. Several repetitive sequences in the U3 region contain GATA, CCAAT, and AATAAA (TATA box) motifs, and the U3 enhancer complex activates the first TATA box in the U3 promoter, causing 25 downstream bases of a specific site to transcribe and synthesize RNA. The second TATA box in the ERV-9 LTR does not act as a poly A signal to terminate RNA transcription, resulting in these synthesized RNAs crossing the R and U5 regions and extending into the downstream genome to activate the transcription of downstream linked genes [108]. In addition, the 5′ end of the upstream locus control region (LCR) of an ERV-9 LTR has been found to regulate the transcription of beta-like globin genes [109]. The ERV-9 LTR in the human genome has strong enhancer activity in embryonic and hematopoietic cells [108,110]. The LTR binds NF-Y transcription factors at the TATA box and recruits the hematopoietic factors MZF1 and GATA-2, which assemble to form the LTR enhancer complex that stimulates the downstream transcription of erythroid progenitor genes. In human erythroid cell lines, ERV-9 LTR-derived lncRNAs acted as stabilizers for ERV-9 LTR enhancer complex assembly, increased the frequency of interaction between the LTR enhancer complex and downstream sites, promoted transcription of bead protein genes, and regulated erythropoiesis in vitro [70]. Together, ERV-derived lncRNAs regulate the erythropoietic network; they are not only involved in physiological regulation, but also have an impact on cancer development (Table 1).

## 4. ERV-Derived lncRNAs Involved in Carcinogenesis

### 4.1. Hepatocellular Carcinoma

Hepatocellular carcinoma (HCC) is a type of liver cancer. It is the third leading cause of cancer death worldwide, and the major causative factors are HBV, HCV, and alcohol [136,137,138]. Most studies on HCC have focused on protein-coding genes, while the ncRNA transcriptome of tumor tissues has been less explored. Hashimoto et al. used the Cap Analysis of Gene Expression (CAGE) method to identify the transcription start sites (TSSs) of ncRNAs in mammals with HCC and found that most of the ncRNAs were derived from the LTRs. The dysregulation of ncRNA expression at the transcriptional level was found to be associated with the development of HCC. HCC patients with high ncRNA expression generally had the clinical characteristics of poor differentiation, aggressiveness, and a high risk of recurrence [139]. ERV LTR-derived ncRNAs have some influence on the development of HCC. However, the specific molecular mechanism is largely unknown.

**Figure 4:**
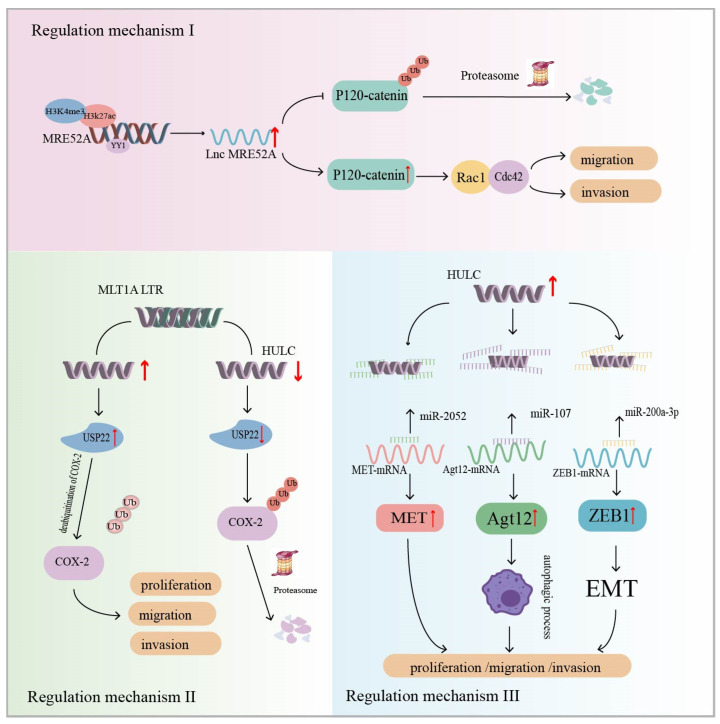
Regulatory mechanism I: Modification of H3K4me3 and H3K27ac histones as well as binding of the nonhistone protein Yin Yang-1 (YY1), a transcription factor, affect *MER52A* transcription. *MER52A* inhibits ubiquitination/proteasome-dependent degradation of p120-catenin, resulting in a prolonged p120-catenin half-life. In turn, p120-catenin induces the activation of Rac1/Cdc42, which regulates HCC cell migration and invasion. The regulatory mechanism II: ERV-derived lncRNA *HULC* increases the expression level of Ubiquitin-specific peptidase 22 (USP22), resulting in reduced ubiquitin-mediated degradation of cyclooxygenase-2 (COX-2) protein. COX-2 expression is upregulated and half-life is prolonged, leading to enhanced growth and metastasis of HCC cells (USP22 is a deubiquitinating enzyme). The regulatory mechanism III: ERV-derived lncRNA *HULC* acts as molecular sponge for miRNAs to promote HCC (MET is a tyrosine kinase associated with metastasis in tumorigenesis; Atg12 is a key regulator of the autophagy process; EMT is epithelial–mesenchymal transition). The black pointed arrow represents positive regulation, while the black flat arrow represents inhibitory regulation; The red up arrow indicates upregulation of expression, while the red down arrow indicates downregulation of expression.

Wu et al. analyzed 10 paired HCC tumor tissue and non-tumor tissue samples and found that ERV1 LTR-derived lncRNA (lnc*MER52A*) was highly expressed in HCC, but not in normal liver cells. Patients with high lnc*MER52A* had advanced TNM stage, less differentiated tumors, and shorter overall survival. In addition, in vivo and in vitro experiments have shown that lnc*MER52A* contributes to the migration and invasion of cancer cells (Table 1). Regarding its specific mechanism, the transcriptional expression of lnc*MER52A* in cancer tissues is subject to modification by two histones, H3K4me3 and H3K27ac, as well as binding by the transcription factor YY1. The overexpression of lnc*MER52A* was characterized by the following aspects. Firstly, lnc*MER52A* regulated the epithelial–mesenchymal transition (EMT) signaling pathway, and the cell morphology was spindle-shaped or fibroblast-like in character. Secondly, lnc*MER52A* inhibits ubiquitination/proteasome-dependent p120-catenin degradation, resulting in the prolonged half-life and increased stability of p120-catenin. Finally, p120-catenin induces the activation of Rac1/Cdc42, which regulates the migration and invasion of HCC cells [115] (Figure 4: Regulatory mechanism I). The study revealed that lnc*MER52A* affects HCC cell migration and invasion through the p120-catenin/Rac1/Cdc42 signaling pathway, which can be used as a biomarker for HCC patients.

A study identified a novel lncRNA, *HULC*, that is highly specifically upregulated in blood and tissues of HCC, by constructing a gene library and with the help of cDNA arrays. Notably, the first exon of the *HULC* gene is mainly composed of the LTR (Table 1) [140]. Xiong et al. reported that lncRNA *HULC* affects HCC development by promoting the stability of the COX-2 protein. Ubiquitin-specific peptidase 22 (USP22) is a deubiquitinating enzyme. The overexpression of *HULC* in HCC cells increased the expression level of USP22, leading to a reduction in the ubiquitin-mediated degradation of COX-2 proteins, resulting in the upregulation of COX-2 expression and half-life prolongation. Tumor cells were more susceptible to growth and metastasis (Figure 4: Regulatory mechanism II) [127]. Several studies have now reported that *HULC* functions as a ceRNA that can act as a molecular sponge for miRNAs in cells. Zhang et al. reported that *HULC* interacts with miR-2052 in HCC cells to upregulate the expression of the miR-2052 downstream target MET. MET is a tyrosine kinase associated with tumorigenesis and metastasis [141]. Studies have confirmed that *HULC* promotes HCC progression through the miR-2052/MET axis in vitro and in vivo [128]. In addition, *HULC* is a molecular sponge for miR-107, which attenuates the binding of miR-107 to Atg12 3′-UTR. Atg12 is a key regulator of the autophagy process. The *HULC*/miR-107/Atg12 axis was found to regulate cellular autophagic activity and may serve as one of the pathways by which *HULC* regulates cell migration and invasion [129]. *HULC* can also isolate the miR-200a-3p signaling pathway to upregulate ZEB1 and enhance EMT to promote HCC migration and invasion (Figure 4: Regulatory mechanism III) [130]. These studies suggest that LTR-derived lncRNA *HULC* influences HCC development. 

lncRNAs not only are important biomarkers of carcinogenesis, but also correlate with the acquisition of resistance to chemotherapeutic treatments after HCC development. linc-*ROR* is a stress lncRNA that is highly expressed in HCC cells and mainly enriched in extracellular vesicles (EVs) (Table 1). Transforming growth factor β (TGFβ) can stimulate signaling pathways associated with antitumor drug resistance [142]. Interestingly, linc-*ROR* has been reported to be involved in mediating TGFβ resistance to chemotherapy. After TGFβ promotes the release of linc-*ROR* from EVs, linc-*ROR* inhibits cancer cell apoptosis by regulating the p53-dependent signaling pathway, which serves as a mediator of TGFβ resistance to HCC chemotherapy [111]. These studies support the importance of ERV-derived lncRNAs in HCC.

### 4.2. Breast Cancer

**Figure 5:**
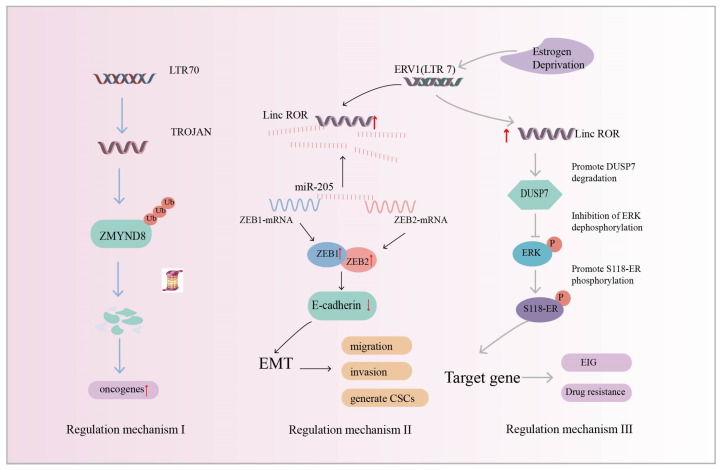
Regulatory mechanism I (Blue Arrow): HERV LTR70-derived lncRNA *TROJAN*. *TROJAN* promotes triple-negative breast cancer (TNBC) progression by degrading ZMYMD8 through the ubiquitination–proteasome system (ZMYMD8 inhibits the expression of genes associated with cancer metastasis). Regulatory mechanism II (Black Arrow): ERV-derived linc-*ROR* acts as a molecular sponge to upregulate the transcription factors Zinc finger E-box-binding homeobox 1 (ZEB1) and Zinc finger E-box-binding homeobox 2 (ZEB2), inducing epithelial–mesenchymal transition (EMT) to promote breast cancer development and metastasis. Regulatory mechanism III (Grey Arrow): linc-*ROR* reduces the stability of dual-specificity phosphatase 7 (DUSP7) and protects extracellular signal-regulated kinase (ERK) from dephosphorylation. Activated ERK promotes Ser118-ER phosphorylation and leads to the upregulation of downstream target gene expression, affecting estrogen-independent growth (EIG) and drug resistance. The red up arrow indicates upregulation of expression.

Breast cancer is one of the most common malignant tumors among women [143], of which triple-negative breast cancer (TNBC) is one of the most aggressive molecular subtypes. Chemotherapy is currently one of the most dominant clinical tools, but has poor results [144]. Increasing evidence suggests that HERVs play a key role in breast cancer disease [145,146,147], and studying the pathogenic mechanism of HERVs in breast cancer can accelerate the development of targeted drugs. HERV transcript levels in TNBC and normal breast tissues were analyzed using transcriptomics and the findings revealed that the HERV LTR70-derived lncRNA, *TROJAN*, which was highly expressed in TNBC tissues, was associated with poor patient survival (Table 1). In addition, *TROJAN*-deficient mice had smaller tumor volumes and metastasis suppression. *TROJAN* affects the migratory and proliferative potential of TNBC and drives tumorigenesis and development [116]. ZMYMD8 has been reported to inhibit the expression of genes associated with cancer metastasis [148]. Strikingly, *TROJAN* degrades ZMYMD8 through the ubiquitination–proteasome system, which, in turn, regulates the expression of downstream oncogenes, leading to the increased migration and invasion of TNBC cells, poorer patient prognosis, and the promotion of breast cancer progression (Figure 5: Regulatory mechanism I) [116].

In addition to HERV-derived *TROJAN* being associated with breast cancer development, Hou et al. found that (ERV1) LTR7-derived linc-*ROR* expression was upregulated in breast cancer samples and may be involved in breast cancer development. Cells with a high expression of linc-*ROR* showed the following three characteristics. Firstly, the cells were spindle-shaped, the epithelial markers almost disappeared, and the mesenchymal markers were significantly upregulated. These results suggest that linc-*ROR* is an inducer of EMT and may be involved in breast carcinogenesis. Secondly, linc-*ROR* promoted breast cancer cell migration and invasion in vitro and tumorigenesis and metastasis in vivo. Finally, the generation and self-renewal of cancer stem cells (CSCs) were increased [112]. Mechanistically, the transcription factors ZEB1 and ZEB2 inhibit promoter activity and control the transcription of epithelial marker E-cadherin, which promotes the EMT program and tumor metastasis [149,150]. miRNAs can silence gene expression at the post-transcriptional level. For example, miR-205 targets ZEB1 and ZEB2 in breast cancer and downregulates their expression [151]. lncRNAs act as molecular sponges and are important regulators of the let-7 microRNA family [152]. linc-*ROR* acts as a miR-205 molecular sponge, preventing the degradation of ZEB1 and ZEB2 and inducing EMT, which is closely associated with tumorigenesis and metastasis [112] (Figure 5: Regulatory mechanism II). The results show that linc-*ROR* is a predictor of breast cancer occurrence and a promising biomarker of breast cancer prognosis.

In addition to the above-reported linc-*ROR*-induced EMT in breast cancer, Peng et al. found that linc-*ROR* regulates estrogen-independent growth (EIG) and drug resistance in breast cancer through the MAPK/ERK signaling pathway. Estrogen deprivation induces linc-*ROR* expression. The phosphatase DUSP7 acts as a negative regulator of linc-*ROR*-mediated ERK activation. The overexpression of linc-*ROR* decreases the stability of the DUSP7 protein, which, in turn, activates the MAPK/ERK signaling pathway to protect ERK from dephosphorylation. Activated ERK promotes Ser118 ER phosphorylation and leads to the upregulation of ER target gene expression. In contrast, the knockdown of linc-*ROR* in estrogen-independent breast cancer cells inhibited cell growth and reduced sensitivity to chemotherapy treatment (Figure 5: Regulatory mechanism III). This suggests that linc-*ROR* acts as a regulator to influence the estrogen-independent growth (EIG) and drug resistance of breast cancer [113]. It has been reported that linc-*ROR* is also an important regulator of human tumor occurrence and development, such as hepatocellular carcinoma [111] and pancreatic cancer [114]. 

### 4.3. Melanoma

Melanoma is a skin cancer caused by malignant tumors of melanocytes [153,154]. The accumulation of mutated genes can lead to carcinogenesis by affecting and altering cell proliferation, differentiation, and death programs [155]. Most melanomas are associated with mutational activation of the *BRAF* gene, and missense mutations of *BRAF* have been reported to be the oncogenic form in approximately 70% of melanomas, compared to a low frequency of mutations in other human cancers. The *BRAF* gene encodes rapidly accelerated fibrosarcoma (RAF) proteins, which are serine/threonine kinases composed of three isoforms, ARAF, BRAF, and CRAF [156]. Mutant *BRAF* activates various signaling pathways such as MAPK, ERK, and Akt, which affect the proliferation, migration, and differentiation of melanoma cells [157,158,159]. 

*BRAF* mutations have been reported to alter gene expression levels in cancer tissues and enhance their association with underlying disease. *BRAF* induces the lncRNA *BANCR* to be overexpressed in metastatic melanoma samples (Table 1). *BANCR* regulates melanoma cell migration, and the knockdown of *BANCR* inhibits melanoma cell migration. In addition, the knockdown of *BANCR* downregulated the chemokine CXCL11, and when CXCL11 was reintroduced, tumor cell migration was restored [160]. Li et al. reported that *BANCR* also promotes proliferation in malignant melanoma by regulating MAPK pathway activation [117]. The above studies suggest that *BANCR* affects the migration and proliferation of melanoma. In addition, *BANCR* is also dysregulated in a variety of cancers, including liver, lung, and colorectal cancer, and is associated with poor patient prognosis [118]. The ERV-derived lncRNA *BANCR* is important for the future study of human cancers.

**Figure 6:**
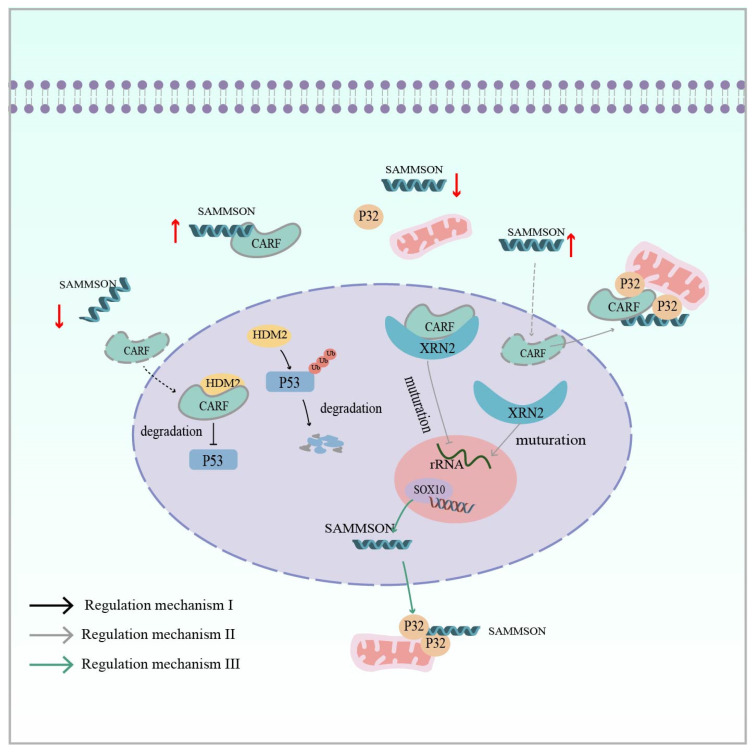
Regulatory mechanism I (Black Arrow): lnc *SAMMSON* in melanoma isolates the CARF protein in the cytoplasm, preventing CARF from interacting with nuclear HMD2 and leading to the ubiquitinated degradation of p53 (CARF is an RNA-binding protein). Regulatory mechanism II (Grey Arrow): lnc *SAMMSON* disrupts the nuclear CARF–XRN2 interaction and CARF enters the cytoplasm to form a complex with p32, which synergizes mitochondrial and cytoplasmic protein synthesis to promote melanoma cell growth. Regulatory mechanism III (Green arrow): SOX10 (transcription factor) targets *SAMMSON*, and transcribed lnc *SAMMSON* interacts with p32, increasing its mitochondrial targeting and contributing to melanomagenesis. The red up arrow indicates upregulation of expression, while the red down arrow indicates downregulation of expression.

Furthermore, lncRNA *SAMMSON* (survival-associated mitochondrial melanoma-specific oncogenic non-coding RNA), promoted by an isolated LTR1A2 element, plays an indisputable oncogenic role in melanoma (Table 1) [69,119]. Its encoding gene is located on chromosome 3p13–3p14, which also contains the melanoma-specific oncogene MITF [161]. Han et al. reported that *SAMMSON* not only affects melanoma growth and survival, but also serves as a novel mediator of adaptive resistance to prevent the apoptosis of melanoma cells induced by RAF inhibitors. Regarding its mechanism in the ERK pathway, the overexpression of *SAMMSON* can act as a molecular sponge to isolate CARF in the cytoplasm, preventing it from entering the nucleus where it interacts with HMD2, leading to the degradation of p53 by ubiquitination. Conversely, when *SAMMSON* is silenced, CARF translocates to the nucleus to bind to HMD2 and inhibit p53 activation through proteasomal degradation (Figure 6: Regulatory mechanism I) [120]. CARF affects p32 in addition to its involvement in p53 regulation. Vendramin and colleagues found that CARF, as an RNA-binding protein, can bind to XRN2 in the nucleoplasm to restrict nucleolus rRNA maturation. In melanoma, the overexpression of *SAMMSON* into the nucleus disrupted the CARF–XRN2 interaction, allowing CARF to enter the cytoplasm to form a complex with p32. This not only promotes nucleolar rRNA maturation, but also increases the synthesis rate of cytoplasmic protein and mitochondrial protein (Figure 6: Regulatory mechanism II) [121]. Interestingly, Leucci also found that the melanoma-specific transcription factor SOX10 targets *SAMMSON* and that transcribed *SAMMSON* binds to the mitochondrial metabolism regulator p32 to enhance mitochondrial targeting and cancer-promoting functions (Figure 6: Regulatory mechanism III) [119]. In addition to melanoma, *SAMMSON* expression is upregulated in several other cancers, such as hepatocellular carcinoma, breast cancer, and glioblastoma, and also influences cancer development [122,123,124].

Overall, the ERV LTR-derived lncRNAs *BANCR* and *SAMMSON*, which are specifically expressed in cancer cells, are biomarkers for melanoma and could be potential targets for cancer therapy.

### 4.4. Other Cancers

In recent years, ERV-derived lncRNAs have become increasingly important in cancer. In addition to the three cancers discussed in detail above, lncRNAs have broad significance in the following cancers.

Pancreatic cancer (PC) is one of the fatal malignancies [162]. linc-*ROR* plays a key role in the development and metastasis of malignant tumors including PC. linc-*ROR* upregulates ZEB1, which then induces EMT to promote the invasive biological behavior of PC. The overexpression of linc-*ROR* in a mouse model promoted the proliferation, migration, invasion, and distant metastasis of pancreatic cancer cells [114]. In addition, a study reported that a lncRNA *EVADR* transcribed from the MER48 ERV element was specifically activated in colorectal tumors (Table 1). The lncRNA was significantly upregulated in cancer tissues compared to normal tissues [125]. Strikingly, it has been noted that *EVADR* was also highly expressed in lung, gastric, and pancreatic adenocarcinomas and is also specifically, rather than universally, activated by the MER48 LTR element [125]. It is biologically important to further investigate whether *EVADR* can be characterized as a candidate biomarker for adenocarcinoma.

Colorectal cancer is the fourth leading cause of cancer deaths worldwide with nearly 900,000 deaths per year [163]. One study identified a lncRNA, *PURPL*, derived from the ERV1 LTR MER61C driver in colorectal cancer (Table 1). The targeted depletion of *PURPL* resulted in a significant increase in p53 transcriptional activity, defective cell growth in vitro, and impaired xenograft tumor growth in vivo. MYBBP1A is involved in the regulation of p53 protein stability, and *PURPL* binds MYBBP1A in the cytoplasm to inhibit the formation of the MYBBP1A-p53 complex [126]. Not only does *PURPL* positively regulate p53, but p53 can also reverse-induce *PURPL* to make it absent, thereby increasing sensitivity to chemotherapy [164]. Interestingly, *HULC* not only affects the occurrence of HCC, but also has a role in colorectal cancer. In colorectal cancer, *HULC* interacts with EZH2, leading to the inhibition of the transcription of the target NKD2, which, in turn, activates the WNT signaling pathway to promote carcinogenesis [131]. Studies have shown that the autoregulatory feedback loop composed of lncRNA *PURPL* and p53, as well as *HULC*, influences colorectal carcinogenesis.

Prostate cancer remains a major medical problem for men. In particular, advanced metastatic cancers are virtually incurable even with high-intensity multimodal treatments [165]. An early study characterized a lncRNA, *SChLAP1*, which is highly expressed in some prostate cancer patients and was found to be critical for cancer cell invasion and metastasis. Regarding its functional mechanism, SWI/SNF mediates chromatin remodeling, which is essential for regulating gene expression, while *SChLAP1* antagonizes the SWI/SNF complex (Table 1) [132]. It has been shown that *SChLAP1* promotes prostate cancer development and may serve as a predictor of poor clinical outcomes.

Bladder cancer is the most common malignant tumor of the urinary tract and one of the most prevalent cancers worldwide. Surgery and chemotherapy have become the mainstay of clinical treatment, and it is important to further explore biological mechanisms [166]. *UCA1* is an ERV-transcribed lncRNA in bladder cell lines, with partial sequence overlap with LTR elements of the ERV1 family (i.e., LTR7 and HERVH) (Table 1). *UCA1* was found to be expressed at high levels in tissues during fetal development, but transcription is silenced in most tissues after birth. In adults, its aberrant activation is associated with the development of cancer. In vitro experiments have shown that high *UCA1* expression increases bladder cancer cell migration and invasiveness, tumorigenicity, and drug resistance [133]. *UCA1* regulates CREB through a PI3K-AKT-dependent signaling pathway, which, in turn, regulates cell cycle progression. A high expression of *UCA1* inhibits the PI3K-AKT pathway, resulting in decreased CREB expression and consequent cell cycle arrest [167].

Ovarian cancer is one of the most common malignancies in gynecology. The early signs and symptoms are not obvious, leading to diagnosis at an advanced stage, at which time there are limited therapeutic options [168]. Rangel et al. screened five SAGE tags specifically expressed in ovarian cancer using the serial analysis of gene expression (SAGE) database and named them human ovarian-cancer-specific transcripts (*HOSTs*) (Table 1). *HOSTs* have been experimentally confirmed to be barely expressed in normal tissues, but upregulated in all subtypes of ovarian cancer [134]. *HOST2* is of most interest to us, with a genome that contains multiple copies of retroviruses and has no obvious open reading frame. lncRNA *HOST2* is derived from the full length of HERV-E and is transcriptionally driven by a flanking LTR2B element [69]. It has been reported that specific and highly expressed *HOST2* promotes the migration, invasion, and proliferation of ovarian cancer tumor cells. The microRNA let-7b is a potent tumor suppressor. *HOST2* lncRNA can act as a sponge to segregate microRNA let-7b and inhibit its function, which, in turn, promotes the expression of the oncogene [135].

## 5. lncRNA-Based Targeted Therapies

The dysregulation of ERV-derived lncRNA transcripts has been associated with cancer development [169,170]. It has also been implicated in a variety of complex biological processes, such as immune regulation [171] and nervous system development [172]. Information about lncRNAs and their functions in diseases is constantly being decoded, which provides a theoretical basis for disease treatment as well as a wealth of potential candidate targets for drug design. Therapies targeting lncRNAs have only become a focus of research in the last decade; however, Winkle et al. reported that no therapies targeting lncRNAs are currently in clinical development [173].

There are several approaches currently in vogue for targeting lncRNAs. The first is small interfering RNA (siRNA), which is complementary to the target lncRNA and recruits arginine-containing RNA-induced silencing complexes (RISCs) to induce the degradation of lncRNAs, mainly in the cytoplasm. This approach has been successfully applied in a variety of preclinical models. A study reported that the lncRNA CASC9 was significantly upregulated in esophageal squamous cell carcinoma (ESCC) species. Different CASC9 sites were targeted with siRNAs, and CASC9-2 and CASC9-3 were found to be the most efficiently knocked down. CASC9-2 siRNA was packaged into lentiviral vectors and ESCC cells were infected with the packaged lentivirus; subsequently, attenuated invasive and migratory capacity was observed [174]. However, the challenge of siRNA off-target effects remains [175]. With the use of siRNA technology, research into siRNA drugs targeting lncRNAs may enter a new era. 

The second approach involves antisense oligonucleotides (ASOs), chemically synthesized short single-stranded oligonucleotides that act primarily in the nucleus. The mechanism of action of ASOs can be divided into two categories: one is that they can bind to complementary RNAs and recruit RNase H, which triggers RNA degradation and alters the expression of downstream proteins; the other is based on spatial site-blocking, whereby ASOs bind to key regions of mRNAs, thereby affecting mRNA maturation or translation into proteins [176]. It has been reported that *TROJAN* affects the migration and proliferative potential of TNBC and is associated with low patient survival [116]. Jin et al. designed eight ASO-transfected MDA-MB-231 LM2 cells, which decreased *TROJAN* expression and diminished cell proliferation. In addition, the expression of *TROJAN* in lung metastatic nodules of ASO-treated mice was significantly lower than that of PBS-treated mice, and the number of lung metastatic nodules was significantly reduced in the treated group [116]. Taken together, the results suggest that the modification of the lncRNA *TROJAN* via ASO interference, a preclinical model, provides a theoretical basis for the treatment of clinical TNBC patients. Therapies for ASOs have led to clinical breakthroughs in recent years. As of 2024, a total of 12 ASO drugs have been commercially approved worldwide in the United States and the European Union, with the majority of indications focused on genetic disorders, mainly targeting mRNAs, and a large number of ASO-based drugs in clinical trials [176,177].

Another preclinical model is CRISPR/Cas9 for dual-localized lncRNAs or lncRNAs with unknown cellular localization. The lncRNA *TTTY15* is highly expressed on the Y chromosome, and in experiments to confirm whether *TTTY15* affects the phenotype of prostate cancer cells, the successful knockdown of *TTTY15* using the CRISPR/Cas9 method resulted in the reduced proliferation and migration of prostate cancer cells [178]. Based on in vitro and in vivo preclinical data, CRISPR/Cas9 remains a potential therapeutic tool. The in vivo delivery of CRISPR/Cas9 is immature compared to in vitro/ex vivo systems. Therefore, unless we can prevent off-target effects and address systemic delivery and other potentially intractable technical issues, clinical applications will remain elusive. Despite the numerous preclinical applications of targeted lncRNAs, further technological advances are needed for actual use in the clinic.

## 6. Summary and Outlook

In this review, we first discussed the factors that influence ERV activation and the role of activation in human health and disease. lncRNAs play important gene regulatory roles, and in terms of their origins, ERVs were found to be a major contributor to the origin, diversification, and regulation of lncRNAs compared to other types of transposable elements. We then focused on how ERV-derived lncRNAs exert biological functions, including antiviral immune modulation, pluripotency maintenance, and erythropoiesis. In addition, they play important roles in cancer development, and we summarized the mechanisms of lncRNA action in different cancers. Although ERV-derived lncRNAs are still poorly understood, their potential mechanisms in tumor growth and progression may guide the development and investigation of new treatments for cancer. However, this also brings new challenges: where is the balance between ERV-derived lncRNAs in physiological regulation and disease? For example, linc-*ROR* is not only associated with the maintenance and differentiation of pluripotent stem cells, but also plays a role in diseases such as breast and pancreatic cancer. In the future, we can explore how ERV-derived lncRNAs can be expressed in specific tissues in terms of ERV activation to exert positive biological functions and inhibit diseases.

## Figures and Tables

**Table 1:** Functional bidirectionality of ERV-derived long non-coding RNAs.

Functional Bidirectionality	lncRNA	Functional Type	TE Type	References
Physiological regulation	lnc-ALVE1-AS1	Antiviral innate immune response	ALVE1	[94,95]
	lnc-LTR5B	Antiviral response	ERV-L LTR5B	[96]
	lnc-EPAV	Antiviral innate immune response	ERV1 LTR	[86]
	lncRNA	Erythropoiesis	ERV-9 LTR	[70,108,109]
	HPAT5	Pluripotency regulation	HUERS-P1 ERV and SINE	[99]
	lncRNA	Pluripotency regulation	HERVH LTR	[33]
	linc-ROR	Pluripotency regulation	(ERV1)LTR7	[102,103]
Carcinogenesis	linc-ROR	HCC chemotherapy resistance	(ERV1)LTR7	[59]
		Hepatocellular carcinoma		[111]
		Breast cancer		[112,113]
		Pancreatic cancer		[114]
	lncMER52A	Hepatocellular carcinoma	(ERV1)MRE52A	[115]
	TROJAN	Triple negative breast cancer	HERV LTR70	[116]
	BANCR	Melanoma, others	(ERV1)MER41B LTR	[117,118]
	SAMMON	Melanoma, others	(ERV1)LTR1A2	[69,119,120,121,122,123,124]
	EVADR	Adenocarcinomas	MER48 ERV	[125]
	PURPL	Colorectal cancer	(ERV1)MER61C LTR	[126]
	HULC	Hepatocellular carcinoma, colorectal cancer	(ERVL-MaLR)MLT1A LTR	[127,128,129,130,131]
	SChLAP1	Prostate	(ERV1)LTR12C	[132]
	UCA1	Bladder cancer, others	(ERV1)LTR7C	[133]
	HOST2	Ovaries	(ERV1)LTR2B	[69,134,135]
